# Managing Osteoarthritis Pain in Underrepresented Populations: Insights from Mexico and Latin America

**DOI:** 10.3390/jcm15062396

**Published:** 2026-03-21

**Authors:** Mónica Vázquez-Del Mercado, Patricia Anaid Romero-García, Pallavi Bhattaram, Carlos Edgardo Mendoza-Díaz, Sergio Ramirez-Perez

**Affiliations:** 1 Instituto de Investigación en Reumatología y del Sistema Músculo-Esquelético (IIRSME), Centro Universitario de Ciencias de Salud (CUCS), Universidad de Guadalajara, Guadalajara 44340, Jalisco, Mexico; 2 Departamento de Biología Molecular y Genómica, Centro Universitario de Ciencias de Salud (CUCS), Universidad de Guadalajara, Guadalajara 44340, Jalisco, Mexico; 3 División de Medicina Interna, Servicio de Reumatología, Hospital Civil Dr. Juan I. Menchaca, Guadalajara 44340, Jalisco, Mexico; 4 Área de Columna Vertebral, Unidad de Especialidades Ortopédicas (Ueso), Hospital Puerta de Hierro Andares, Zapopan 45118, Jalisco, Mexico; 5 Emory Musculoskeletal Institute, Emory University School of Medicine, Atlanta, GA 30329, USA; 6 Department of Orthopaedics, Emory University School of Medicine, Atlanta, GA 30322, USA; 7 School of Medicine, International Program, Universidad Autónoma de Guadalajara, Zapopan 45129, Jalisco, Mexico

**Keywords:** osteoarthritis management, Mexico and Latin America, pain relief strategies, healthcare disparities, personalized medicine, multidisciplinary healthcare models

## Abstract

Osteoarthritis (OA) has emerged as a significant public health crisis in Latin America, with prevalence rates in Mexico doubling over the last two decades. Despite this growing burden, current management in underrepresented populations —defined here as groups facing structural barriers to care, including rural or remote communities, uninsured individuals, and socioeconomically disadvantaged groups —is hindered by a critical mismatch between international guidelines and regional healthcare realities. This narrative review, synthesized under the SANRA framework, evaluates the last 20 years of evidence to address the structural and clinical barriers that sustain a 50% diagnostic gap at the primary care level and high rates of inadequate pain relief. Using Mexico as a primary case study, we move beyond conventional symptomatic treatment to explore the complex interplay of central sensitization, neuroinflammation, and metabolic phenotypes, factors often overlooked in standardized protocols. By identifying the limitations of current pharmacological reliance and the underutilization of non-pharmacological interventions, this work proposes a strategic shift toward a multidisciplinary, patient-centered model. We outline a translational roadmap that integrates multi-omic research with personalized therapeutic strategies, emphasizing the need for evidence-based clinical practice guidelines tailored to the socioeconomic and genetic contexts of Latin American patients. Ultimately, this review serves as a call to action to bridge the healthcare disparity gap, offering a framework for innovative, integrative care to transform long-term clinical outcomes in developing healthcare systems.

## 1. Introduction

Osteoarthritis (OA) is the most common form of arthritis affecting the elderly population [1,2,3]. The most recent reports have indicated that worldwide prevalence rates have dramatically increased by 113.25% over the past 20 years [4]. The highest prevalence rate has been observed in Latin America, which increased by 203.56% from 1990 to 2019 [4]. OA significantly impacts the global economy through direct healthcare costs and healthcare-unrelated costs due to the increasing number of primary healthcare visits, decreased labor productivity, and poor quality of life [5,6,7,8,9]. The healthcare-related costs include primary care visits, hospitalization, and medicines, while the major non-healthcare-related cost is productivity losses due to absenteeism [6]. In 2020, OA was the seventh most significant cause of 70-year-old adults living with disabilities [10]. OA pain severity has also been associated with several comorbidities such as obesity, hypertension, osteoporosis, and emotional distress such as anxiety and depression [9,11,12]. Those critical factors affecting the OA burden are strongly associated with inadequate chronic pain management and long-term disability. Inadequate management might be influenced by several aspects, such as variation in pain intensity, frequency, and pattern, along with the unstandardized tools for its evaluation in OA patients [13,14]. Another contributor to inadequate pain management is ineffective OA therapies for alleviating pain sensitization [15,16]. Nociceptive pain mechanisms are typically described in OA, which are mediated by interactions between the central and peripheral systems [17,18]. Both systems seem to activate specific nociceptors at the joint by mechanical, thermal, and chemical stimuli [19]. The prevalence of OA has dramatically increased in Latin America, particularly in the Mexican population, over the last two decades, and the forecasted values for 2050 show a worst-case scenario [4,10]. Additionally, heightened pain sensitivity (i.e., a low pain threshold to mechanical stimuli) has been identified as a relevant risk factor associated with OA in the Mexican population [20], opening avenues for identifying and stratifying high-risk patients. This narrative review aims to provide a critical and comprehensive overview of OA pain management in Mexican patients, focusing on challenges faced and potential opportunities in research and interventional programs. In this context, underrepresented OA populations include groups with limited access to timely diagnosis and multimodal pain care (e.g., uninsured/underinsured individuals, rural/remote communities, and socioeconomically disadvantaged groups). Given the scarcity of locally generated evidence across the region, we use Mexico as a case study and integrate Latin American evidence when it meaningfully informs implementation challenges or interventions not studied locally.

## 2. Methodology

This review was conducted according to the Scale for the Assessment of Narrative Review Articles (SANRA) [21]. A structured search strategy was conducted in PubMed and Scopus (using equivalent terms across databases) to identify evidence published between 1987 and 2024. We applied an extended timeframe due to the limited number of original studies addressing OA-related pain in Mexico. Eligible publications were in English or Spanish to capture the relevant literature produced in Mexico and Latin America. Search terms combined OA-related concepts (e.g., “osteoarthritis”/OA), pain constructs (e.g., pain intensity, pain phenotypes/sensitization, and pain management), and geographic terms (Mexico/Latin America), complemented by contextual keywords on burden and epidemiology (Supplementary Table S1). Given the narrative (non-exhaustive) purpose of this review and the iterative nature of topic refinement, including backward and forward citation screening, we did not aim to provide PRISMA-style record accounting; instead, we provide an explicit search framework and eligibility criteria (Supplementary Table S1) to ensure transparency and reproducibility.

Duplicates were removed using EndNote. Initial screening was performed on titles and abstracts, followed by full-text assessment. Screening was conducted independently by the first and corresponding authors, and discrepancies were resolved in consultation with the second author. Two predefined eligibility layers were applied: (i) for evidence mapping of original Mexican studies, we included original studies (prospective/retrospective studies, cross-sectional studies, clinical trials, and preclinical models) and excluded systematic/narrative reviews, as well as secondary OA and post-anterior cruciate ligament surgery OA to improve clinical homogeneity; (ii) for the overall narrative, we included original studies, clinical trials, systematic reviews, meta-analyses, and narrative reviews reporting pain- and/or function-related outcomes in Mexican OA populations and, when relevant, Latin American populations. Global evidence was included only to contextualize mechanisms or interventions not locally studied. Studies without pain/function outcomes, populations outside the scope without justification, non-primary reports, and conference abstracts were excluded.

To enhance transparency specifically for the evidence-mapping layer, we provide a simplified selection roadmap with counts (from records identified to final included studies) in Supplementary Figure S1, which documents the selection process for the original Mexican studies included in Table 1. Evidence was synthesized narratively across thematic sections addressing burden, pain management approaches and challenges, and research opportunities and future directions.

## 3. OA Burden in Latin America and Mexico

**Figure 1:**
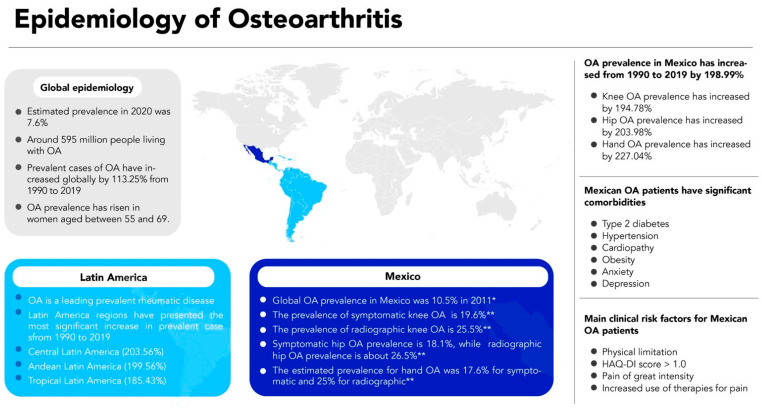
Overview of OA epidemiology in Mexico. Increased and Latin American OA prevalence has been observed. The prevalence of OA in Mexico varies depending on the state. However, a global percentage increase was reported from 1990 to 2019. Several comorbidities have also been associated with OA prevalence in the Mexican population. The main clinical risk factors for Mexican patients with OA are physical limitation, great-intensity pain, and increased use of pain therapies. * A multicenter study evaluated five distinct regions across the country. ** Studies were restricted to a specific region of central Mexico and would not be generalized to the general Mexican population.

The prevalence and incidence of radiographic and symptomatic OA have been reported in several populations, indicating the increased ratio associated with age [4,10,22]. The estimated worldwide prevalence for OA in 2020 was 7.6%, representing around 595 million people living with OA [10]. In addition, prevalent cases of OA increased globally by 113.25% from 1990 to 2019 [4]. The global OA prevalence rose remarkably in women aged between 55 and 69 in 2019. The study also reported that Latin America regions, including the Central (203.56%), Andean (199.56%), and Tropical (185.43%) regions, have presented the most significant increase in prevalent cases. It is important to note that for all three regions, knee, hip, and hand OA (i.e., OA affecting hand joints) prevalence increased by 2 folds compared to the overall OA prevalence [4]. Although OA is more prevalent in elderly populations (>70 years old), the reports also indicated that younger adults between the ages of 25 and 49 might develop OA [10]. Moreover, an analysis performed of the forecasted percent change between 2020 and 2050 has revealed that the age-standardized prevalence will dramatically increase for knee, hand, and hip OA in the Central Latin American region [10]. Prevalence of rheumatic diseases was assessed in eight Latin American indigenous communities from Argentina, Venezuela, and Mexico; the results indicated that OA is a leading prevalent rheumatic disease [23]. The analysis also showed that OA was significantly associated with diabetes mellitus type 2, hypertension, cardiovascular diseases, and disability evaluated by a Health Assessment Questionnaire Disability Index (HAQ-DI) score > 0.8 [23]. These observations suggest a critical global rising issue to be addressed for older and younger adults with OA (Figure 1), particularly in those individuals from Latin America.

Symptomatic and radiographic OA have been evaluated in distinct Latin American populations, revealing essential differences in the reported prevalence [23,24,25,26]. On the one hand, the prevalence of symptomatic knee OA in Latin America ranges from 1.55% in Peru to 19.6% in Mexico [24,27,28]. On the other hand, the prevalence of radiographic knee OA, defined according to the Kellgren and Laurence score as grade ≥ 2, has been found to be high in Brazil and Mexico with an estimation of 22% and 25.5%, respectively [24,27,29]. Similar findings were observed for the hip OA prevalence, which was higher in Mexico (symptomatic, 18.1%; radiographic, 26.5%) in comparison with Peru (0.37%) [24,27,28]. The estimated prevalence reported in Mexico for hand OA was 17.6% for symptomatic and 25% for radiographic OA [27]. However, it is important to note that those studies were restricted to a specific region of central Mexico and would not be generalized to the general Mexican population. In this regard, a multicenter study performed in 2011 evaluated the global OA prevalence in Mexico and reported an estimated value of 10.5% by evaluating five distinct regions [30]. Even though estimated OA prevalence could vary due to genetic, cultural, educational, and socioeconomic factors across the country [31,32,33,34,35,36], OA remains the most prevalent rheumatic disease in the Mexican population (Figure 1).

In the same line of evidence, overall OA prevalence increased in Mexico from 1990 to 2019 by 198.99%, where knee, hip, and hand OA showed a prevalence increase of 194.78%, 203.98%, and 227.04%, respectively [4]. Additionally, the age-standardized prevalence in the Mexican population reported in 2020 indicated that knee (4570.5 per 100 000) and hand (3011.2 per 100 000) OA rates are higher than the estimated global prevalence (knee OA = 4307.4 per 100 000; hand OA = 2226.1 per 100 000) [10]. Another multiregional study in Mexican patients evaluated the socioeconomic, lifestyle, and clinical factors associated with OA [20]. Their results indicated that OA patients have more significant comorbidities such as type 2 diabetes, hypertension, cardiopathy, obesity, anxiety, and depression. Interestingly, the main clinical risk factors for OA patients are physical limitation (OR = 1.6), HAQ-DI score > 1.0 (OR = 3.3), pain of great intensity (OR = 2.6), and increased use of therapies for pain (OR = 4.3) [20].

A comparative analysis carried out in Latin America described the critical situation of patients with OA. Overall findings showed that the mean duration of knee pain was 6 years, predominantly affecting the patella–femoral joint, and females were primarily affected by the disease [37]. The presented scenario in Mexico was alarming due to the prevalence of moderate pain in OA patients; the results also reported that the highest percentage of unemployed patients was observed in Mexico. Additionally, Mexican patients presented higher physical limitations than the other Latin American patients included in the study, and regrettably, almost 95% of Mexican patients neither had access to any institutional or socialized medical care system nor had health insurance coverage [37].

These reports strongly suggest that the prevalence of chronic pain is significantly high in OA and uncover the importance of designing, implementing, and performing research, clinical trials, and interventional programs focused on managing pain in Mexican individuals with OA.

## 4. OA Pain Management in Mexico

**Figure 2:**
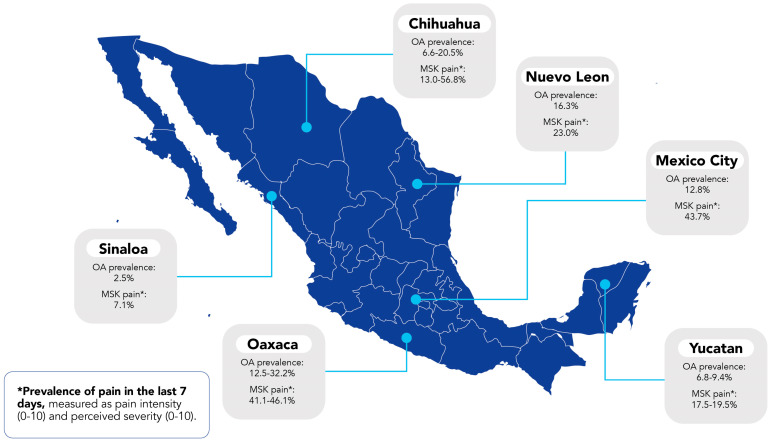
Prevalence of OA and musculoskeletal (MSK) pain across the country. The map of Mexico presents the OA prevalence in the studied states according to the reports published by the Community Oriented Program for the Control of Rheumatic Diseases (COPCORD), an initiative to measure and evaluate pain and disability in rheumatic disorders. The OA prevalence of each state is reported in percentages below the state name, and the percentage of MSK pain is reported as a percentage or percentage range below the “MSK pain” label. NOTE: This figure is intended as an illustrative synthesis; COPCORD-based estimates were obtained from selected states and may differ by sampling frame and methodology, thus limiting direct comparability across states.

Even though only a few studies have evaluated OA-related pain in Mexican patients, the evidence has indicated a significant role of pain with disability (Figure 2). A study performed in the southeastern region of Mexico reported that the prevalence of non-traumatic musculoskeletal (MSK) pain was 19.6%, primarily affecting knees and hand joints [31]. The study also reported that OA was the most prevalent rheumatic disease in the southeastern population of Mexico [31]. In another study carried out in the northeast region of Mexico, it was reported that pain defined as a visual analog scale (VAS) ≥ 4 was significantly associated with treatment selection in OA patients, with NSAIDs and analgesics (acetaminophen) being the most prescribed [36]. Moreover, VAS pain ≥ 4 was identified as a critical risk factor associated with OA treatment (OR = 1.9; 95%CI, 1.2–2.8; *p* = 0.002) [36], which makes pain a relevant factor and predictor for seeking medical attention, particularly in OA patients.

Hand OA and RA coexistence has also been assessed in Mexican patients [38]. An observational study reported that patients with RA and concomitant hand OA had higher pain intensity than patients without hand OA (*p* = 0.003). Furthermore, pain intensity significantly correlated with high disease activity (r = 0.69, *p* = 0.001) and functional disability (r = 0.58, *p* = 0.001) [38]. Another study in Mexican patients found that erosive hand OA (EHOA) was associated with higher pain sensitization and functional impairment, evaluated through the VAS and Australian/Canadian osteoarthritis hand index (AUSCAN) pain subdomain [39]. In addition, the VAS positively correlated with hand functional disability evaluated by the Stanford Health Assessment Questionnaire (HAQ). A cross-sectional, multi-center, and observational study assessed the impact of inadequate pain relief (IPR) on distinct categories, including quality of life, function, and limitation in Mexican patients with knee and hip OA [40]. Their results indicated that 50.2% of OA patients were categorized as having IPR. Interestingly, patients with IPR were also significantly older than patients without IPR (66.8 vs. 62.5 years, respectively); IPR positively correlated with a high body mass index (BMI) and obesity. The most commonly prescribed treatments were NSAIDs (84.4%) and paracetamol (33.2%), which were unable to show differences in IPR; however, OA patients treated with opioids (15.6%) showed a high percentage of IPR [40]. Additionally, the Euro-QoL five dimensions (EQ-5D) instrument, evaluated for the Latin American region was used to assess general health status, showing that patients with IPR reported moderate to severe problems with mobility, severe pain, and finally, severe anxiety and depression [40]. One additional study evaluated health-related quality of life (HRQoL) among distinct chronic diseases in a Mexican population; the results revealed that OA had the highest VAS values compared to RA, diabetes mellitus, and end-stage renal disease [41]. Moreover, disability evaluated by the HAQ-DI showed a higher value compared to the RA group [41]. These findings strongly suggest that pain and disability remain key concerns regarding OA pathology among Mexican patients.

## 5. A Twenty-Year Overview of OA Pain Management in Mexican Patients

Inadequate management of OA-related pain in elderly patients is associated with significant disability and poor quality of life and impacts the socioeconomic burden of both patients and the government. Physicians and rheumatologists have taken steps to manage OA-related pain in Mexican and Latin American patients through several strategies; a summary of them is discussed in this review (Table 1).

**Table 1:** OA pain management in Mexican patients over the past 20 years.

Clinical Trials					
Therapeutic Approach	Therapeutic Agent	No. of Patients Receiving the Intervention	Study Design	Relevant Data	Ref.
Intramuscular pharmacological therapies	Glycosaminoglycan–peptide complex	25 KOA	Randomized, double-blind, placebo-controlled, parallel-group trial	Significant improvements were seen in pain at rest (*p* < 0.05), pain on walking (*p* < 0.05), and morning stiffness (*p* < 0.05) over the 2-year follow-up period.	[42]
	Tiaprofenic acid	31 KOA 206 OA	Multicenter open trial Open trial	Significant reductions in swelling degree, pain intensity scores, and functional limitation values were observed in patients with OA (*p* < 0.01).	[43]
	Sodium diclofenac and vitamin B complex	48 KOA	Double-blind, randomized clinical trial	The combination of diclofenac and vitamin B complex significantly reduced VAS pain scores with respect to the baseline values and when compared with diclofenac alone (*p* < 0.05).	[44]
Intraarticular pharmacological therapies	Polymerized type I collagen	27 KOA	Prospective, randomized, double-blind placebo-controlled clinical trial	Lequesne index and WOMAC subscales for pain, stiffness, and disability significantly decreased at 12 months of treatment compared to those of the placebo group (*p* < 0.05).	[45]
	Polymerized type I collagen	10 KOA	Randomized, double-blind, and placebo-controlled clinical trial	Lequesne index and WOMAC subscales for pain and disability significantly decreased from baseline to 6 months of treatment (*p* < 0.01).	[46]
	Polymerized type I collagen	309 KOA	Open-label, single-center study	WOMAC subscales for pain, stiffness, and disability significantly decreased from baseline to 60 months of treatment (*p* < 0.05).	[47]
	Polyvinylpyrrolidone collagen Hylan G-F 20	70 KOA 40 KOA	Retrospective, randomized, longitudinal, and observational clinical trial	Both hylan G-F 20 and collagen-PVP decreased WOMAC pain scores after treatment administration. The study did not report statistical analysis.	[48]
	Hyaluronic acid	89 KOA	Prospective, randomized, open clinical trial	WOMAC subscales for pain, stiffness, and disability significantly decreased from baseline to 12 months of treatment and compared to those of the group treated with betamethasone (*p* < 0.0001).	[49]
	Sodium bicarbonate and calcium gluconate	73 KOA	Randomized, double-blind, parallel-design clinical trial	Lequesne index and WOMAC subscales for pain, stiffness, and disability significantly decreased from baseline to 12 months of treatment (*p* < 0.05).	[50]
	8-amino-7-oxononanoate synthase (BioF2)	8 severe KOA	Prospective, randomized, 3-arm, parallel-group clinical trial	WOMAC subscales for pain, stiffness, and disability significantly decreased from baseline to 12 months of treatment compared to those of the group under NSAIDs (*p* < 0.05).	[51]
	8-amino-7-oxononanoate synthase (BioF2)	119 KOA	Prospective, randomized, 2-arm parallel-group phase III clinical trial	Pain VAS scores significantly decreased and PASS was increased at 12 months of treatment compared to those of the group under NSAIDs (*p* < 0.001).	[52]
Selective inhibitors of cyclooxygenase 2	Celecoxib	40 OA	Randomized double-blind clinical trial	Pain VAS scores significantly decreased from baseline to 6 weeks of treatment (*p* < 0.05).	[53]
	Etoricoxib	97 OA	Open-label multicentric study	Pain VAS lower scores represent a significantly decreased pain/stiffness and better functioning (*p* < 0.0001).	[54]
Non-pharmacological interventions	Low-intensity pulsed ultrasound	10 KOA	Observational clinical trial	Pain decreased (*p* = 0.005), and the Lequesne severity index diminished (*p* = 0.008).	[55]
	Isokinetic exercise	33 KOA	Quasiexperimental study	Muscle strength improved (*p* = 0.04), and pain intensity decreased (*p* = 0.01) compared with the control group (isometric exercise).	[56]
	Kinesio taping	16 KOA	Single-center, single-blind, randomized clinical trial	Pain significantly decreased from baseline to week 6 (*p* = 0.01). No significant differences were observed in pain subscales compared to those of the placebo group.	[57]
	Long-term home-based therapies	15 TMJ OA	Experimental pre–post intervention study	The pain significantly decreased (*p* = 0.001), and TMJ functionality significantly improved (*p* = 0.001)	[58]
**Preclinical Models**					
**Therapeutic Agent**	**Experimental Model**	**Relevant Data**			**Ref.**
S(+) and R(-) enantiomers of flurbiprofen	PIFIR model	Intravenous administration of S(+)-flurbiprofen was 100-fold more potent than R(-)-flurbiprofen to produce an antinociceptive action on Wistar rats.			[59]
NSAIDs and opioids	PIFIR model	Female Wistar rats were orally administered distinct combinations of ketorolac plus tramadol. Three combinations of ketorolac + tramadol (1.0 + 17.78, 1.78 + 10, and 1.78 + 17.78, mg/kg, respectively) produced the maximum antinociceptive effects.			[60]
Rofecoxib and tramadol	PIFIR model	Female Wistar rats were orally administered distinct combinations of rofecoxib plus tramadol. Five combinations of rofecoxib + tramadol exhibited fewer antinociceptive effects compared to the sum of the effects produced by each drug alone. Rofecoxib alone (17.8 mg/kg) showed a more antinociceptive effect (46.8%) than the combination.			[61]
Flavonoids and diterpenes	Kaolin/carrageenan-induced OA model	Oral administration of diterpenes kingidiol (10 mg/kg) reduced the ankle thickness and significantly decreased the levels of TNF-α, IL-1β, IL-6, and IL-17 in the synovial capsule of kaolin/carrageenan-induced OA mice.			[62]

Abbreviations: OA, osteoarthritis; TMJ, temporomandibular joint; WOMAC, Western Ontario and McMaster Universities Osteoarthritis Index; VAS, visual analog scale; NSAIDs, non-steroidal anti-inflammatory drugs; TNF-α, tumor necrosis factor alpha; IL, interleukin; PIFIR, pain-induced functional impairment; PASS, Patient Acceptable Symptom State; PVP, polyvinylpyrrolidone. Note: This table summarizes original evidence from Mexico for mapping purposes; study designs and methodological quality vary, and results should not be interpreted as equivalent across RCTs, open-label/observational studies, and preclinical models.

### 5.1. Intramuscular Pharmacological Therapies

Several clinical trials have focused on evaluating distinct biomolecules and chemical compounds as potential pharmacological strategies for treating OA in Mexican patients. The glycosaminoglycan-peptide complex was one of the first treatments used for the management of knee OA [42]. A clinical trial assessed the clinical outcomes of 50 Mexican patients who received intramuscular injections for 3 years. Significant overall improvements regarding pain at rest, pain on walking, and morning stiffness were observed in the group of glycosaminoglycan-peptide complex treatment versus placebo after the 3-year period [42]. In another study, intramuscular administration of tiaprofenic acid was evaluated in 31 Mexican patients with knee OA; the results indicated that the treatment significantly reduced joint, severe pain, swelling, tenderness, and improved functional limitation after 5 days of treatment [43]; systemic side effects were present only in 2 cases (headache and dizziness). Another clinical trial evaluated an intramuscular combination therapy of sodium diclofenac and vitamin B complex to potentiate the effect on pain relief in 48 patients with severe OA [44]. Pain relief perception was successfully decreased, and a significant number of patients achieved complete pain relief after 12 h of the intramuscular administration of the combination therapy compared to diclofenac alone [44].

### 5.2. Intraarticular Pharmacological Therapies

Two clinical trials evaluated the efficacy and safety of intraarticular injections of polymerized collagen versus placebo in knee OA patients [45,46]. The treatment with polymerized-type I collagen significantly reduced pain intensity, stiffness, and disability evaluated through the VAS, Western Ontario and McMaster (WOMAC) Universities Osteoarthritis and Lequesne indexes, and Lequesne indexes; response to treatment was sustained until three [46] and six [45] months follow-up. Urinary CTX-II increased in the placebo group, indicating the progression of joint damage [45,46]. In addition, immunological profile assessment revealed that polymerized collagen treatment decreases proinflammatory cytokine expression (IL-1β and TNF-α) dependent on circulating CD8+ and CD4+ T cells, and CD14+ monocytes [46]. The long-term treatment with polymerized-type I collagen was further evaluated in a Mexican cohort of 309 knee OA patients, reporting significant clinical improvements in terms of pain intensity and functional disability when data from baseline to 60 months was compared without records of serious adverse effects [47]. In addition, at 60 months, none of the patients who received the intervention underwent total knee replacement (TKR); therefore, the overall data suggest that intra-articular administration of polymerized-type I collagen may be a potentially beneficial therapeutic approach for knee OA.

Intra-articular administration of polyvinylpyrrolidone collagen (PVP) and hylan G-F 20 was evaluated in 110 knee OA patients; both treatments were able to reduce pain intensity [48]. However, the study has significant limitations, as it did not specify the doses, the time intervals between infiltrations, or the time frame between the baseline and the clinical evaluation following treatment administration. Another comparative trial evaluated the efficacy of hyaluronic acid (HA) and betamethasone in knee OA. Patients who received intra-articular injections of HA showed superior effectiveness at 12 months by significantly reducing pain in comparison to patients treated with betamethasone [49]. A study in 51 patients with KOA assessed efficacy and safety after intra-articular sodium bicarbonate and calcium gluconate administration. The study findings indicated that after 12 months of the intervention period, patients showed significant improvements in all WOMAC subscales compared to baseline values (pain: 81% treatment vs. 77% baseline; stiffness: 92% treatment and 79% baseline; physical functioning: 90% treatment vs. 81% baseline); improvements were maintained after 6 months of follow up period, and relatively few adverse reactions were observed [50].

A novel cell-free bioactive compound, 8-amino-7-oxononanoate synthase (BioF2), has been reported to successfully target distinct OA pathogenic hallmarks in Mexican clinical trials by decreasing all WOMAC subscales (pain, stiffness, and functional disability) at 12-month follow-up [51,52]. Intra-articular administration of BioF2 in OA patients showed similar clinical improvements to the TKR OA group, which was taken as a gold standard for severe OA management [51]. BioF2 significantly increased the cartilage volume evaluated through nuclear magnetic resonance after one year of treatment, with no serious adverse effects [51].

### 5.3. Selective Inhibitors of Cyclooxygenase 2

Blocking of pronociceptive actions by selective inhibition of cyclooxygenase-2 (COX-2) has also been addressed in a study that evaluated the efficacy and safety of diclofenac-cholestyramine and celecoxib in Mexican patients with OA. After 6 weeks, both treatments significantly reduced pain evaluated by the VAS scores; the main side effects observed for both treatments were epigastric pain, upset stomach, nausea, and headache [53]. A multicenter study assessed the quality of life and pain relief in Mexican patients with OA, RA, and chronic low-back pain treated with etoricoxib [54]. Findings indicated that etoricoxib significantly decreased pain and improved physical functioning when compared with baseline VAS values in more than 50% of patients. Furthermore, enhancements in patients’ quality of life were observed, particularly in the physical and psychological components, while patients’ satisfaction with etoricoxib treatment reached 92% [54].

### 5.4. Non-Pharmacological Interventions

Distinct interventions have been evaluated at the non-pharmacological level to achieve clinical improvements in OA patients. In this regard, an observational trial assessed low-intensity pulsed ultrasound in patients with second and third-degree KOA [55]. Magnetic resonance imaging (MRI) analysis evaluated cartilage thickness at the end of the intervention; however, no statistically significant differences were observed. On the contrary, low-intensity pulsed ultrasound had a significant therapeutic effect on pain reduction and functionality improvement among OA patients [55]. Another eight-week interventional study evaluated the effectiveness of isokinetic (experimental group) versus isometric (control group) exercises in KOA patients. Evaluation of patients at the end of the study revealed significant improvements in muscle strength and pain relief in the isokinetic exercise group compared with the control group [56], which supports the current recommendations of exercise in clinical guidelines as a therapeutic option for OA management. Similarly, a randomized clinical trial evaluated the effectiveness of Kinesio Taping [9] on pain intensity in knee OA patients [57]. After six weeks of intervention, the experimental group receiving KT showed significant improvements in pain perception evaluated by VAS compared with baseline data; however, when measures were compared with the control group, there were no statistically significant differences in pain improvements neither in VAS nor WOMAC values [57]. Similar exercise approaches have also been considered for temporomandibular joint (TMJ) OA patients [58]. A small pre–post clinical study in patients with TMJ OA evaluated pain intensity (*n* = 15) as the primary outcome before and after an exercise intervention. Their findings indicate that interventions such as intraoral and external massage, along with stretching and strengthening exercises, significantly reduce TMJ pain intensity and improve TMJ mobility and function after 6 months of follow-up [58].

### 5.5. Preclinical Models

Animal models have also been explored to test antinociceptive drugs. A preclinical rat study evaluated the effect of intravenously administered flurbiprofen enantiomers using the pain-induced functional impairment model (PIFIR); the S(+)-flurbiprofen enantiomer showed superior antinociceptive effects [59]. Combination therapy of NSAIDs, e.g., ketorolac and opioids such as tramadol, has also been evaluated as a modulator of arthritic nociception in the PIFIR model [60]. Although ketorolac was more potent than tramadol, showing great antinociceptive efficacy, the combination therapy showed a synergistic antinociceptive effect with moderate gastrointestinal side effects [60]. One more study assessed the combination of rofecoxib plus tramadol in the same PIFIR model; however, the antinociceptive effect of rofecoxib alone was superior and showed less gastrointestinal side effects than the combination therapy [61]. Flavonoids (acacetin, pectolinaringenin, and 6-methoxykaempferide) and diterpenes (kingidiol) obtained from Baccharis conferta have also been shown to produce anti-inflammatory and anti-arthritic effects in an OA model induced by kaolin/carrageenan (K/C) [62]. Oral administration of those active compounds significantly reduces ankle joint thickness and decreases the production of TNF-α, IL-1β, IL-6, and IL-17 in the synovial capsule of mice. In addition, the biological effect of diterpene kingidiol successfully reduced hyperalgesia in the K/C-induced OA model [62]. Another bioactive compound that has shown significant antinociceptive and anti-inflammatory effects is the isothiocyanate, sulforaphane, obtained from Brassica oleracea [63]. Intraperitoneal sulforaphane (0.1 mg/kg) was administered to rats subjected to distinct pain models. The plantar test results for thermal nociception indicated that sulforaphane had a significant antinociceptive effect compared to that in the vehicle group [63]. Inflammation was assessed in a carrageenan-induced edema model; the inflammatory response significantly decreased at 6 h by the effect of sulforaphane administration, and the effect remained until 24 h [63]. Taken together, these findings suggest that biologically active compounds warrant further investigation as potential pharmacological approaches for managing nociception and inflammation in OA pathology.

### 5.6. Alternative and Experimental Treatments

Current treatment options for OA are predominantly pharmacological, aiming to provide symptomatic relief. However, no existing treatment can halt the progression of chondral degeneration or reverse cartilage damage [64]. This gap in the optimal management of OA pain has led alternative treatments to emerge as potential pain relievers in OA. Recent advancements in alternative pain management treatments for OA have become visible, focusing on tissue engineering to regenerate joint chondral lesions and reduce pain. Bone Marrow Stem Cells (BM-SCs) have been proposed as a therapeutic alternative for OA pain management due to their potential regenerative effects on chondral damaged tissue. For instance, a prospective, open-label, phase I/II clinical trial conducted in Mexican patients over 30 years old with KOA assessed the efficacy of a single intra-articular injection of autologous BM-SCs. Compared to those receiving acetaminophen, the BM-SC group showed significant reductions in VAS and WOMAC scales at one week, one month, and six months follow-up, with superior results favoring BM-SC at the six-month mark (*p* < 0.0001 VAS, *p* < 0.001 WOMAC) [64].

Alternatively to BM-SC, autologous leukocyte-poor platelet-rich plasma (LP-PRP) infiltration, containing biologically active cytokines, has been explored as an adjunct option; however, the evidence remains heterogeneous and depends on protocol characteristics and study design. A study on the adult Mexican population with early KOA demonstrated that LP-PRP significantly improved clinical outcomes, reducing VAS and WOMAC scores and enhancing quality of life compared to acetaminophen [65]. PRP has been studied for its ability to reduce the inflammatory response in chondrocytes through the modulation of IL-1β, IL-1Ra, and TGF-β. By inhibiting IL-1β’s inflammatory effects, PRP injections are thought to reduce pain and stimulate chondrocyte activity [66]. A study comparing acetaminophen and three intra-articular PRP injections for early KOA found PRP to be superior in decreasing VAS (*p* < 0.001 vs. the acetaminophen group: *p* < 0.01), WOMAC (*p* < 0.05 for the acetaminophen group and *p* < 0.001 for the LP-PRP group) pain scores, and quality of life at six, twelve-, and twenty-four-months follow-up [66]. Similar studies have compared PRP with other first-line treatment NSAIDs such as celecoxib. A randomized controlled trial with 60 Mexican patients with KOA compared oral celecoxib and a single injection of autologous PRP for early KOA. The study revealed that PRP was significantly more effective than oral celecoxib in reducing VAS and WOMAC scores (*p* < 0.001 for both) after one year [67].

Another randomized controlled, prospective, longitudinal clinical trial assessing the dosage and effectiveness of PRP found that triple PRP injections showed greater therapeutic effects, as measured by VAS and WOMAC scores (*p* = 0.0007 and *p* = 0.0209, respectively), compared to a single PRP injection at a 48-week follow-up [68]. This suggests that the clinical improvements may be due to PRP’s ability to accelerate physiological recovery and reduce the pro-inflammatory and catabolic effects of interleukin-1β [68]. Despite favorable findings, a systematic review and meta-analysis of five clinical trials comparing single versus multiple PRP injections on pain and functionality in KOA patients found that single injections were similarly effective in reducing pain (*p* = 0.48 [95%CI 1.09 to 2.31]). However, multiple PRP injections had a significantly superior impact on knee functionality at six months [69].

A meta-analysis of 31 RCTs comparing PRP effectiveness in patients with early-to-moderate KOA versus end-stage OA suggested that PRP significantly improves function and decreases pain regardless of the Kellgren-Lawrence OA stage [70]. According to this study, pain relief was more pronounced in studies with stages 1–3 and 1–4 OA after PRP therapy (*p* < 0.00001 and *p* > 0.00001) [70]. However, it is important to note the observed heterogeneity in therapeutic schemes (e.g., volume injected, administration technique, number of injections) and in follow-up durations of the assessed studies. Addressing disparities in PRP preparation methodologies, a meta-analysis and systematic review of fourteen clinical trials showed that exogenously activated PRP more effectively improves pain relief and functionality in knee OA patients [71].

Other studies have explored methods aimed at reducing cartilage degeneration and pain in other commonly affected joints, such as the hip. Botulinum toxin type A (BoNT-A) has been proposed as a conservative treatment for hip OA pain. An experimental, longitudinal, open-label study evaluating BoNT-A for pain management in 35 patients (45 hips) with hip OA (Kellgren and Lawrence grades II-IV) reported significant pain reduction after 90 days (VAS *p* < 0.0001), along with improvements in WOMAC scores for pain (*p* < 0.0001), stiffness (*p* < 0.002), and function (*p* < 0.001). Additionally, BoNT-A significantly improved flexion, and internal and external rotation after 90 days (*p* < 0.0001 for all three parameters) [72]. These results suggest that BoNT-A acts as a protease, cleaving the transport vesicle SNAP-25, which affects the cholinergic terminals’ hyperfunctionality in OA. In addition, BoNT-A may impact glutamate and Substance P, potentially inhibiting over-excitation in nociceptive transmission [72].

Other studies have evaluated therapeutic options for post-surgical pain in OA patients. A prospective, randomized, evaluator-blind pilot study involving fifty patients with meniscal lesions and OA compared viscosupplementation with HA and PRP for pain relief after knee arthroscopic debridement. The study found that five intra-articular HA injections post-arthroscopic debridement were more effective (65.20% total WOMAC score reduction) than PRP (55.01%) and conventional care (14.5% increase) in reducing pain at 18 months post-intervention [73]. Researchers suggest that HA may decrease chronic pain, stiffness, and functionality by acting as an inflammation mediator, restricting pain nociceptors, stimulating chondrocyte growth, and reducing apoptosis in damaged cartilage [73].

Although recent preclinical and clinical research has explored these biological approaches, including autologous and allogeneic mesenchymal stem cell products, evidence remains mixed, and comparative data suggest pain outcomes can be similar to standard intra-articular corticosteroids. Given cost, regulatory considerations, and methodological limitations in available studies, these therapies should be considered only in selected settings and ideally within well-designed clinical trials.

### 5.7. Clinical Tools

Several rating scales have been developed and used to assess and classify symptoms of OA. Amongst the most common ones, the WOMAC scale and the knee clinical rating scale are two of the scales most commonly used around the globe; these two scales, together with the Bristol Score, have shown higher scores when assessing validity, consistency, and sensibility when compared to other scales such as Six-Minute Walk and Thirty-Second Stair Climb [74]. Despite the number of clinical scales assessing the affectation degree of KOA and the clinical stage of the patient, significant variability in results has been identified, hampering the reliability of the existent clinical scales [74]. In the Mexican population, through a clinometric, prospective, controlled, observational, transversal, and analytic, a clinical index “índice modificado para el estado clínico de la rodilla con osteoarthritis” (modified index for the clinical status of the knee with osteoarthritis) (MSH1) was developed to assess the functional state of patients with KOA based on the Bristol Score and its items susceptible to improvement. According to this study, the MSH1 scale showed better results in consistency (weighted Kappa 0.81, 90%) and validity when compared to the Bristol Score [74]. These results propose MSH1 as a valuable and reliable clinical index for patients with knee OA [74]. Another study has also proposed a Spanish version of the 12-item Knee Injury and Osteoarthritis Outcome Score (KOOS) as a valid, reliable questionnaire to measure patients’ opinions about their diagnosed KOA and its associated problems [75].

Apart from assessing clinical stage and OA affection degree, some other tools have been developed to increase the diagnosis of OA and other rheumatic disorders in the Mexican population. For instance, the Community Oriented Program for the Control of Rheumatic Diseases (COPCORD) evaluated the COPCORD core Questionnaire (CCQ) performance to diagnose any rheumatic diseases. According to this study, four variables—pain in the last seven days, NSAID treatment, a high pain score, and having a previous diagnosis—resulted in R = 0.24, with *p* > 0.01, suggesting that this questionnaire may be useful in the detection of rheumatic pathologies in community surveys [76].

Besides clinical scores assessing symptoms of OA, imaging and measuring techniques are essential elements for OA research and diagnosis. A cross-sectional, observational study using a Multielgon system (digital goniometer) comparing the Range of Movement (ROM) of the metacarpal and first interphalangeal joints in housewives with nodal hand OA vs. a healthy control group concluded that the Multielgon system might be used for the objective evaluation of function in patients with hand OA [77]. Conventional radiography (plain X-ray) is commonly used as a first-line modality to support diagnosis and grade severity in several peripheral joints (e.g., knee/hand OA joints). However, in anatomically complex joints such as the TMJ, cross-sectional imaging (computed tomography (CT) and MRI) is often preferred for structural assessment. The radiological findings observed in patients with OA, such as osteophytes and reduced joint space, have been demonstrated to be correlated with pain and joint stiffness. However, X-rays present significant challenges due to the complex nature of pain in joints and the subjectivity of pain perception [78]. A case–control study was conducted to identify which radiological findings or models can be used to determine which patients are at greater risk of knee pain. The results indicated that patients with specific changes in knee anatomy, such as medial osteophytes, alterations in the medial joint space width, and chondrocalcinosis, identified by X-rays, are at a higher risk of developing pain compared to patients without radiological OA findings [78].

Besides imaging studies, biomarkers such as the C-terminal telopeptide of type II collagen (CTX-II) play a major role in evaluating joint injury and assessing the degradation severity of cartilage in KOA. Based on the importance of these two diagnostic tools, recent studies have studied the correlation between KOA’s radiographic findings and the affected joint’s clinical state using a combination of specific clinical scores and radiographic scales [79]. In the Mexican population, a cross-sectional descriptive study evaluated 155 northeastern women with KOA, assessed anteroposterior and lateral knee X-rays, together with the WOMAC scale for pain classification, and urine concentrations of the biomarker CTX-II. The results of this study found increased concentrations of the urinary CTX-II biomarker, the WOMAC score for pain degree, and radiographic staging [79]. These results highlight the importance of determining biomarkers as complementary tools for diagnosing and prognosis degenerative disease.

An early diagnosis and the evaluation of risk factors directly impact the efficacy of OA treatment. Nevertheless, there are several shortcomings in the effective management of OA. In order to address the existing therapeutic gaps, a group of researchers proposed a therapeutic integrative model for patients with OA across the three existing levels of the Mexican healthcare system through a prospective pilot, interventional, and clinical study [80]. The model proposed an integrative and multidisciplinary team for the nutritional, physiotherapeutic, social, and psychosocial evaluation of patients with KOA, which was included in the intervention group [80]. The patients included in the intervention group exhibited improvements in the VAS, mobility arcs, body mass index, and psychological items. These results highlight the necessity for developing integrative health programs to enhance clinical outcomes in patients with OA and optimize the allocation of financial resources dedicated to treating such patients [80].

## 6. Key Challenges of Underrepresentation

Building upon the twenty-year evidence mapping presented in Table 1, it is evident that a significant gap exists between clinical research findings and their practical application in the Mexican healthcare system.

As discussed in previous sections of this review, the inadequate management of OA pain, particularly in elderly patients in Mexico, leads to significant disability and poor quality of life, contributing to a substantial socioeconomic burden for both patients and the social security systems. While some preclinical models have evaluated potential therapeutic approaches, there is still a notable absence of clinical trials to confirm these findings. According to the studies presented in Table 1, most pain management approaches in the Mexican population have focused on patients with knee OA. However, hand and hip OA require further investigation due to the dramatic increase in their prevalence in Mexico.

Other current limitations observed in the reviewed studies include the lack of effect on acute-phase reactants, such as C-reactive protein (CRP) down-regulation, along with the absence of standardized structural assessment (e.g., plain radiography for joint space narrowing and severity grading, and, where feasible in research settings, MRI/CT for more detailed structural evaluation). The disconnect between joint pain and radiologically reported structural damage is another limiting factor requiring innovation to better understand multifactorial drivers of OA pain [81,82,83]. These multifactorial contributors could include a combination of synovitis, innervation, and vasculature of the joint capsule, systemic factors, and patient-specific factors, including biological sex, comorbidities, and genetic and epigenetic factors, which likely influence pain perception in OA patients. The situation is exacerbated by a lack of randomized designs and insufficient sample sizes in existing studies, highlighting the urgent need for larger, double-blind clinical trials to validate potential treatment options, particularly for early OA pain.

Another key challenge identified is the role of primary care physicians in diagnosing patients with rheumatic diseases. Often, primary care physicians lack the specialized knowledge necessary for accurate diagnosis, with diagnostic accuracy rates not exceeding 50% [84]. This issue may arise from gaps in knowledge and a lack of confidence in approaching rheumatic patients among general practitioners or non-rheumatologists. This is particularly problematic for the four most common rheumatic diseases, RA, spondylarthritis (SpA), systemic lupus erythematosus (SLE), and OA, which all share joint pain as a common symptom [84]. As previously noted, delayed diagnosis and treatment, along with limited treatment duration, are among the most critical challenges in OA pain management. However, patient-directed alterations in treatment regimens based on symptom severity, side effects, and the growing popularity of traditional medicines are additional factors contributing to unpredictable outcomes and effectiveness in OA pain management [40]. Furthermore, there is limited data characterizing patients in Mexico who continue to experience moderate to severe pain despite analgesic treatment [40]. The possible factors that may contribute to this issue include insufficient or lack of non-pharmacologic interventions, declining treatment efficacy over time, presence of comorbidities, and barriers related to treatment access and healthcare system characteristics [40].

Effective OA pain management may also rely on clearly defined procedural details outlining essential treatment elements, including optimal timing and administration methods. Unfortunately, there are still insufficient descriptions of both pharmacological and non-pharmacological interventions [85]. While systematic reviews have sought to connect current knowledge with clinical practice, translating their findings into evidence-based clinical care poses challenges due to diverse user needs across different countries [85]. Therefore, addressing this critical concern demands the exploration of potential approaches tailored to diverse healthcare contexts globally.

Pharmacological recommendations have facilitated the implementation of treatment protocols in clinical practice. However, there is also significant non-adherence to these recommendations among physicians. A survey assessing adherence to the EULAR 2000 recommendations for knee OA found that only 54% of physicians followed both pharmacological and non-pharmacological guidelines in general practice [86]. Non-adherence to non-pharmacological recommendations was particularly pronounced in the management of patients over 75 years old. Conversely, adherence to recommendations increased among patients with a BMI ≥ 25 kg/m^2^ and when patients expressed preferences for specific treatments [86]. Factors associated with increased physician adherence included regular continuing medical education, years since graduation from medical school, and familiarity with the EULAR 2000 recommendations [86]. These findings highlight the relevance of continuing medical education to perform an adequate patient approach and an effective management of OA pain.

The implementation of certain interventions in clinical practice can be challenging due to conflicting and inconclusive recommendations often found in clinical practice guidelines [87]. A notable example of this occurs in the management of hand OA, where there is inconsistency regarding the recommendations for acetaminophen and intra-articular corticosteroid injections. Hence, this therapeutic recommendation was offered as the last option for the proposed algorithms [87]. Translating knowledge into evidence-based clinical care poses significant challenges due to diverse healthcare contexts and structural limitations. To address this, we provide a strategic framework that aligns the eight key challenges identified in this review with international guidelines and local implementation tiers, which is synthesized in the translational roadmap (Table 2). This approach moves beyond descriptive study summaries toward scientific reasoning and actionable clinical guidance for underrepresented settings.

## 7. Future Perspectives for Developing Countries

Future directions are required to identify and create strategies for the most suitable management of OA pain (Table 2). Based on the key challenges identified in this review, as an initial attempt, we propose implementing innovative therapeutic approaches in Mexican OA patients that have already been applied in developed countries with promising results. In this regard, an innovative initiative performed by eighteen Ibero-American countries aimed to design an algorithm for optimizing their diagnosis of patients with joint pain in four rheumatic diseases, including OA [84]. The items evaluated for OA encompassed symptoms, signs, and paraclinical tests. The algorithm considers joint pain as a common semiological point for four rheumatic diseases (RA, SpA with peripheral involvement, SLE, and OA) and was designed for primary care physicians to facilitate the approach of the patient and diagnosis accuracy. This innovative tool allows non-rheumatologist physicians to improve their clinical approach to identifying patients with joint pain [84]. Similar strategies have been implemented for the management of OA, where user-friendly algorithms developed from clinical practice guidelines enhance clarity for the physician, improve the patient’s approach, and perform a more accurate diagnosis of OA [87]. While international benchmarks from EULAR, ACR, and OARSI provide high-quality evidence [88,89,90,91], their implementation must be adapted to local “reality checks” such as limited access to specialized orthoses and specialists in rural regions (Table 2). Future research and policy should prioritize these implementable core care packages —focusing on simplified education and home-based exercise— over costly, unproven interventions, such as stem cell or PRP injections, which should remain restricted to standardized research protocols.

From a pragmatic perspective, primary care settings in Mexico and Latin America may also benefit from a brief, feasible assessment set to guide phenotype-oriented management. This includes combining pain intensity scales (VAS/NRS) with functional tools validated in Spanish versions, for instance, the 12-item Knee Injury and Osteoarthritis Outcome Score (KOOS-12) [75]. When pain is disproportionate to structural findings, screening for neuropathic-like features and central sensitization becomes essential. Tools such as the Douleur Neuropathique 4 (DN4), validated in Mexican clinical cohorts [40], and the Spanish version of the Central Sensitization Inventory (CSI) [92], can distinguish between nociceptive, neuropathic, and nociplastic pain phenotypes. In practice, identifying a sensitized phenotype should trigger a shift in therapeutic decision-making: rather than escalating traditional NSAIDs, clinicians should prioritize multimodal care, including structured exercise and evidence-based centrally acting agents like duloxetine, as recommended by international guidelines for cases with central pain processing involvement [88,89].

As an additional pharmacological approach, the use of opioids has been proposed for the treatment of chronic OA pain in Latin America [93]; however, the limited evidence available evaluating long-term opioid therapy in Mexican patients —where a study reported a high percentage of inadequate pain relief (IPR) among OA patients treated with opioids [40]— raises concerns about the implementation of such interventions. Despite the barriers to prescribing opioids and their moderate availability in Mexico, there are still some critical concerns regarding the risk factors for developing prescription drug abuse and addiction [93]. Although the short-term use of opioids is recommended for the treatment of OA in the manual of opioids for pain treatment in Latin America [94], certain individual aspects must be considered for each patient, such as pain severity, past medical and family history of drug abuse, individualized dose, assessment of effectiveness and adverse effects, the patient’s written consent, and importantly, a multidisciplinary evaluation must be performed [93]. Given that IPR is observed in half of the Mexican OA population, a cautious approach to opioids is mandatory [40]. In accordance with the consensus established in Table 2, oral and transdermal opioids are strongly discouraged for routine care, positioning them as a specialist-only option reserved for end-stage cases with rigorous risk monitoring and clear stopping rules [88,89].

Despite the challenges, recent preclinical and clinical research has identified several promising strategies that may be explored as potential treatment modalities for mitigating the pain associated with both symptomatic and radiographic OA. In this regard, autologous and allogeneic mesenchymal stem cells have been at the forefront of experimental therapies for pain relief in the short term [95,96,97]; however, when evaluating intra-articular injections of various mesenchymal stem cell sources compared to corticosteroids as the gold standard, knee pain scores were similar [98]. This contrasting evidence highlights the fact that although mesenchymal stem cell products are safe, future research is essential to identify the cell source, the cell product, and the responding patient population for these therapies to outperform the current standards of OA treatments. We anticipate that future clinical trial study designs will hugely be inspired from the basic science and pre-clinical data from animal models. For instance, targeting specific subsets of pain-associated fibroblasts and chondrocytes identified from single-cell transcriptomics from OA as well as RA patients has the potential to yield novel targets for pain-inhibiting therapeutics with essential joint protective capabilities [99,100,101,102,103]. Other studies have suggested several molecular targets, such as the NGF [104], BDNF [105], CCL2/CCR2 [106,107,108], and CCL17 [109,110], for mitigating nociceptive joint pain due to OA.

Regarding non-pharmacological therapies, exercise is universally recommended as a primary strategy for the management of KOA pain and could be a valuable and cost-effective tool for OA patients in Mexico [111]. However, the current literature does not provide indisputable evidence either for or against the causal relationship between exercise and improved OA pain [112,113,114,115,116]. Nonetheless, combining exercise with intraarticular mesenchymal stem cell therapy or other biological or non-biological drugs has shown promise in pre-clinical studies; hence, the need for designing clinical trials and systematically testing these modalities remains paramount [117,118]. Another potential cost-effective intervention may be the use of probiotics to combat joint pain [119,120]. This approach is supported by recent basic and pre-clinical research findings on the role of intestinal microbiome dysbiosis in OA [121,122]. In this regard, a number of clinical and preclinical studies testing the effectiveness of probiotics concluded that Lactobacillus strains might be useful for managing pain and inflammation in OA [123,124,125,126,127].

**Table 2:** Translational roadmap for OA management: integrating international guidelines with research priorities and implementation realities in Mexico and Latin America.

Challenges in OA Pain Management	Future Directions (Research Priorities)	Guideline-Aligned Recommendations (Hierarchy Anchor)	Reality Check & Actionable Status (Mexico & LatAm)
1. Inadequate pain management in elderly Mexican patients (disability & poor quality of life).	Develop and integrate comprehensive strategies to overcome barriers, improve diagnosis accuracy, and enhance treatment effectiveness.	Strongly Recommended: Education, self-management, and structured land-based exercise [88,89,91].	Reality Check: High prevalence of DM/hypertension and obesity in Mexico complicates oral NSAID use [20]. Status: Implementable (Primary Care): Core care package (education and home exercise).
2. Focus on knee OA; research gap in hand & hip OA.	Conduct larger, double-blind clinical trials focusing on hand and hip OA to establish validated treatment protocols.	Hand OA: First CMC joint orthoses (strong); topical NSAIDs (first-line) Hip OA: Core care package (strong) [88,89,90].	Reality Check: Scarcity of custom orthoses and occupational therapists or specialists in rural areas [128,129]. Status: Specialist-Dependent: Use of prefabricated orthoses in primary care and implementation of hybrid telerehabilitation.
3. Lack of clinical trials to confirm preclinical findings; lack of X-ray analysis.	Explore innovative therapies (biologics, epigenetic modifiers, and stem cell-based treatments). Conduct trials with pain and X-ray as outcomes.	Strongly Against: Stem cell injections and biologics for routine care due to heterogeneous evidence [88,89].	Reality Check: High out-of-pocket costs (up to 15% of income) and lack of standardized protocols in private practice [130]. Status: Research-Only: Restricted to standardized clinical trials only.
4. Primary care physicians’ lack of specialized knowledge (low diagnostic accuracy <50%).	Enhance education and training for primary care physicians to improve referral accuracy.	Core Principle: Diagnosis based on clinical assessment; imaging not always required for patients > 45 years [89,91].	Reality Check: A total of 69% of local CPG recommendations are difficult to implement in primary settings [131]. Status: Implementable (Primary Care): Use of simplified clinical decision aids and validated short tools like KOOS-12 and DN4.
5. Patient-directed alterations and use of traditional medicine.	Implement evidence-based guidelines that address conflicting recommendations for non-pharmacological interventions.	Strongly Recommended: Shared decision-making to align patient expectations and improve adherence [88,91].	Reality Check: Cultural reliance on complementary and alternative medicine (CAM) use and need for culturally adapted diets [132,133]. Status: Implementable (Primary Care): Culturally sensitive counseling.
6. Non-adherence to treatment guidelines by physicians.	Provide CME to improve adherence to pharmacological and non-pharmacological guidelines.	Reinforcement Principle: Education must be an ongoing intervention reinforced at subsequent clinical encounters [91].	Reality Check: Only 26% of Mexican IMSS patients receive exercise advice; 5% get paracetamol as first-line treatment [131]. Status: Implementable: Audit-feedback and resource-tiered algorithms.
7. Conflicting recommendations in hand OA management.	Develop and implement evidence-based guidelines to resolve conflicting recommendations.	Consensus Core: Topical NSAIDs prioritized; hand orthoses for 1st CMC; oral NSAIDs for limited duration [88,90].	Reality Check: Conflicts in acetaminophen and intra-articular steroid use across guidelines hinder standard protocols [87]. Status: Specialist-Dependent: Referral for inflammatory phenotypes.
8. Limited evidence on long-term opioid use for chronic OA pain in Latin America and Mexico.	Research the long-term efficacy and safety of opioid therapy. Integrate risk-mitigation and addiction strategies.	Strongly Against: Oral/transdermal opioids. Conditional: Tramadol only for persistent cases [88,89]	Reality Check: IPR was observed in 50% of Mexican OA patients despite treatment [40]. Status: Specialist-Only: Reserve for end-stage cases with strict monitoring.

Abbreviations: CME, Continuous Medical Education; CMC, Carpometacarpal; CPG, Clinical Practice Guideline; DM, Diabetes Mellitus; IMSS, Instituto Mexicano del Seguro Social; IPR, Inadequate Pain Relief; LatAm, Latin America; NSAIDs, Non-Steroidal Anti-Inflammatory Drugs; OA, Osteoarthritis.

## 8. Conclusions

Despite significant advances in OA research and treatment, substantial gaps remain in the effective management of OA-related pain, particularly in developing countries such as Mexico. The main challenge is not the absence of international guidance, but the limited availability and uneven implementation of guideline-concordant, multimodal pain care for diverse and underrepresented populations. Restricted access to timely diagnosis, rehabilitation services, and coordinated multidisciplinary care contributes to inadequate pain relief and amplifies the burden of chronic pain.

At the same time, increasing insights into mechanisms such as central sensitization and neuroinflammation support a shift toward phenotype-informed and more personalized management. Pharmacological interventions (e.g., NSAIDs and intra-articular injections) can provide symptomatic relief but remain insufficient to modify long-term disease trajectories. Biologic injectables (e.g., platelet-rich plasma and mesenchymal stem cell products) show heterogeneous and mixed evidence and should be considered only in selected settings and ideally within standardized clinical trials, especially in resource-limited contexts. Non-pharmacological strategies, including exercise and physical therapies, are foundational but remain inconsistently implemented by patients and clinicians. High-prevalence comorbidities (e.g., obesity and diabetes) further complicate management, underscoring the need for integrated, multidisciplinary care.

Future directions should prioritize pragmatic, adequately powered randomized trials and implementation-focused studies in Mexico and Latin America to validate context-adapted therapies and scalable care models that combine pharmacological and non-pharmacological approaches. Developing resource-tiered pathways that distinguish implementable primary-care actions from specialist-dependent and research-oriented strategies will be essential to reduce disparities and improve pain outcomes and quality of life in underrepresented OA populations.

## Data Availability

Non-applicable.

## Supplementary Materials

The following supporting information can be downloaded at https://www.mdpi.com/article/10.3390/jcm15062396/s1. Supplementary Table S1. Conceptual design of the literature search and eligibility criteria with a synthesis structure and a Mexico/Latin America–adapted guideline-to-practice roadmap. and Supplementary Figure S1. Simplified literature selection roadmap for evidence mapping original Mexican studies.
