# Supplementary material for: Managing Osteoarthritis Pain in Underrepresented Populations: Insights from Mexico and Latin America
Source: J Clin Med. 2026 Mar 21;15(6):2396. doi: 10.3390/jcm15062396 (PMC13026629; doi:10.3390/jcm15062396)
Supplement: Supplementary file 1 [file jcm-15-02396-s001.zip › Supplementary Figure S1.pdf]

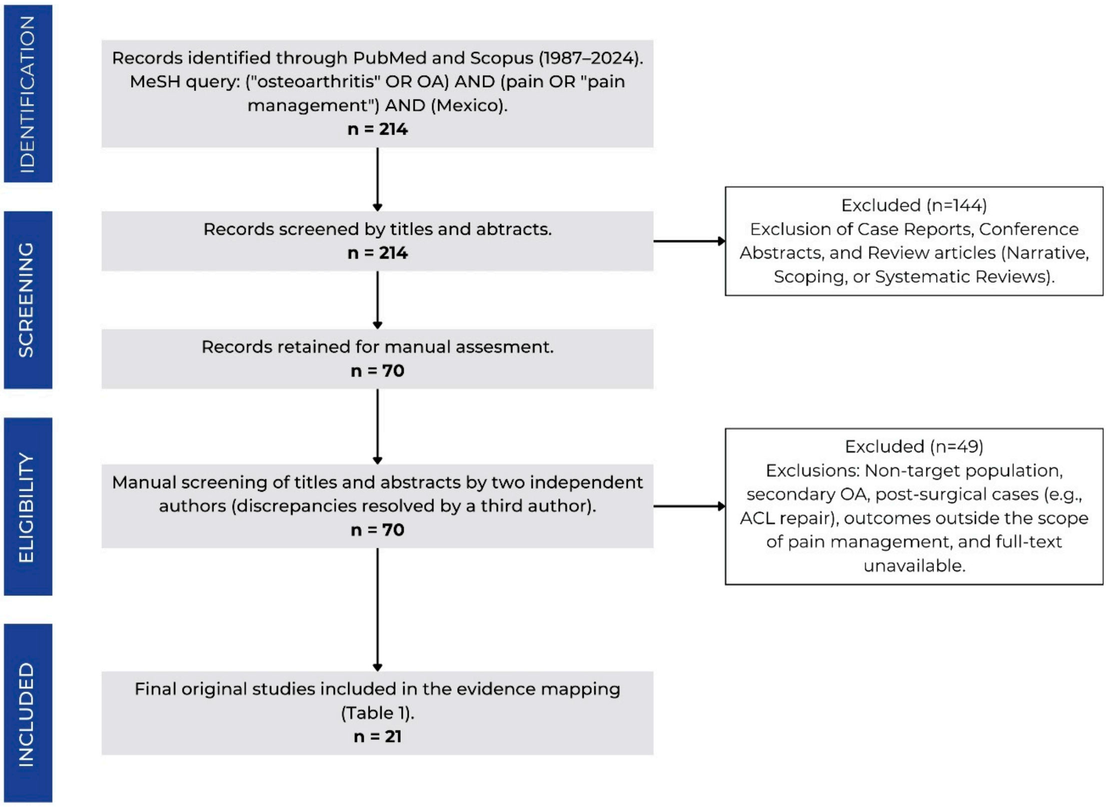


Narrative review (SANRA framework). Schematic illustrates evidence mapping of original Mexican studies.

**Supplementary Figure S1.** Simplified literature selection roadmap for evidence mapping original Mexican studies.
