# Supplementary material for: Managing Osteoarthritis Pain in Underrepresented Populations: Insights from Mexico and Latin America
Source: J Clin Med. 2026 Mar 21;15(6):2396. doi: 10.3390/jcm15062396 (PMC13026629; doi:10.3390/jcm15062396)
Supplement: Supplementary file 1 [file jcm-15-02396-s001.zip › Supplementary Table S1.pdf]

Supplementary Table S1. Conceptual design of the literature search and eligibility criteria with a synthesis structure and a Mexico/Latin America–adapted guideline-to-practice roadmap.

Search items	Details
**Databases**	PubMed; Scopus (equivalent terms adapted per database).
**Publication years**	1987-2024 (extended timeframe due to limited original evidence on OA-related pain in Mexico).
**Language**	English; Spanish.
**Core search domains (keywords/terms)**	<u>Condition:</u> osteoarthritis OR OA.<br><u>Pain constructs:</u> pain, pain management, pain sensitization/central sensitization, pain phenotypes.<br><u>Geography:</u> Mexico OR Latin America/Latin American.<br><u>Contextual:</u> burden, prevalence, epidemiology.
**Example PubMed query (adapted per database)**	(“osteoarthritis” [MeSH Terms] OR osteoarthritis OR OA) AND (pain OR “pain management” OR “pain sensitization” OR “central sensitization” OR phenotype) AND (Mexico OR “Latin America” OR Latin American).
**PIO strategy**	Adults with osteoarthritis in Mexico/Latin America; pharmacological and non-pharmacological pain management strategies; pain and function-related outcomes.
**Deduplication**	Duplicates removed using EndNote.
**Screening process**	Title/abstract screening followed by full-text assessment. Screening performed independently by the first author and the corresponding author; discrepancies resolved with the second author.
**Eligibility (Table 1: evidence mapping of original studies)**	**Included:** original studies (prospective/retrospective, cross-sectional), clinical trials, preclinical models reporting pain- and/or function-related outcomes in OA.<br>**Excluded:** systematic reviews and narrative reviews; secondary OA; post-anterior cruciate ligament surgery OA.
**Eligibility (Main narrative text: synthesis and context)**	**Included:** original studies, clinical trials, systematic reviews, meta-analyses, and narrative reviews addressing OA-related pain (pain intensity, pain phenotypes/sensitization, and pain management) in Mexico; Latin America when pertinent; global evidence only for contextualizing mechanisms/interventions not locally studied.
**General exclusions (both layers)**	Studies without pain/function outcomes; populations outside the scope without justification; non-primary reports; conference abstracts.
**Data extraction domains**	Study design; country/setting; joint(s) studied; sample size/model; intervention/exposure; pain and/or function outcomes; main finding(s).
**Synthesis approach**	Thematic narrative synthesis aligned to manuscript structure: burden/epidemiology, management strategies, preclinical models, therapeutic challenges, and opportunities/future directions
**Implementable clinical strategies**	Roadmap integrating International Guidelines and Mexico/Latin America realities; applicable recommendations.
